# Current Limitations for the Assessment of the Role of the Gut Microbiome for Attention Deficit Hyperactivity Disorder (ADHD)

**DOI:** 10.3389/fpsyt.2020.00623

**Published:** 2020-06-26

**Authors:** Andreas Hiergeist, Jana Gessner, André Gessner

**Affiliations:** Institute of Clinical Microbiology and Hygiene, University Hospital Regensburg, Regensburg, Germany

**Keywords:** gut microbiome, gut brain axis, attention deficit hyperactivity disorder (ADHD), antibiotics, probiotics

## Abstract

High throughput sequencing of bacterial 16S rRNA genes and metagenomes were applied to analyze complex microbial communities inhabiting the human gut and other body sites, and their role in numerous diseases. Studies in animals were important for elucidating the effects of the gut microbiota on the brain and behavior, and the responsible mechanisms. Recent studies in patients have identified bacterial taxa of the gut microbiome possibly impacting different neurological and psychiatric disorders including ADHD. Furthermore, antibiotic treatment of infections globally shape compositions of gut microbiota and might indirectly influence ADHD development. However, published studies revealed still partially incongruent results. Potential reasons for the still ill defined role of gut microbiota in ADHD comprise a) different study designs b) small number of patients c) different age groups analyzed d) inclusion of only treatment naive patients versus patients under medication e) differences of males versus females ratios and f) the heterogenous technology applied for microbiome sequence analysis. Furthermore, the complex interplay between the gut microbiome and individual host genetic factors requires much larger sample sizes and additional patient genome information. Alternative treatment options like probiotics or dietary interventions for ADHD therapy might offer new opportunities to prevent or treat this increasingly common disease. Clearly, further studies are needed to clarify molecular mechanisms imparting the gut brain axis as basis to modify commensal microbiota or their functions to target ADHD. The purpose of this review is to evaluate the most recent literature on the role of the gut microbiome in ADHD.

## Introduction

Complex microbial communities inhabiting the human body are a major factor influencing the development of several diseases and are the determinant for functionally important pathogen-host interactions. The analysis field of human microbiome science is currently undergoing a period of exponential increase of knowledge. (1, 2).

A functional link between microorganisms of the gut and the brain has long been postulated, but in recent years, reports on causal effects of the gut microbiota on brain functions and behavior, and the underlying molecular mechanisms of the gut-brain-axis have begun to be elucidated.

Animal models implied that stress potentially could directly or indirectly alter the gut microbiome composition and that pathogens of the gastrointestinal tract can affect host behavior. For example, germ-free (GF) mice displayed enhanced hormonal response to stress induced by individual isolation, suggesting that the gut microbes impact the neuroendocrine hypothalamic–pituitary–adrenal (HPA) axis, the central stress response system. Furthermore, GF animals treated with antibiotics showed reduced anxiety-like behavior compared with specific pathogen-free (SPF) controls: GF mice stayed longer in the illuminated compartment of the light–dark box and on the open arms of the elevated plus maze, than their SPF counterparts. In a rat model of anxiety and depression, Schmidtner et al. (3) could recently show that depressive-like, but not anxiety-like behavior, was alleviated by 3 weeks of minocycline antibiotic treatment exclusively in male rats with a concomitant reduction in microglial numbers in the infralimbic and prelimbic prefrontal cortex. Further, minocycline lead to a robust shift in cecal microbial composition accompanied by a reduced expression of proinflammatory cytokines. Interestingly, minocycline markedly increased abundance of Lachnospiraceae, a family of bacteria known for its butyrate production. Accompanying behavioral differences, the brains of animals with absent or altered or gut microbiota display various molecular differences. As reported recently (4) microbiome manipulation in antibiotic-treated or GF mice results in profound alterations of fear extinction learning. Single-cell RNA sequencing from brain areas such as the medial prefrontal cortex showed markedly altered gene expression profiles in excitatory neurons, glia, and other cell types as well as defective remodeling of postsynaptic dendritic spines and diminished neuronal activity in the medial prefrontal cortex. Microbiome reconstitution experiments showed that restoration of normal extinction learning in adulthood is possible in a limited neonatal developmental time period. Concenctrations of four metabolites were found to be markedly reduced in GF animals and were related to neuropsychiatric disorders in patients and mouse models, implying that molecules produced by gut microbes may directly affect brain function and thereby behavior.

## Current Research

Due to a lack of suitable animal models and only few studies with still severe technical limitations in humans relatively little is known about the role of gut microbiota in the pathogenesis of ADHD and molecular mechanisms potentially involved have not been defined so far. As of December 2019 and starting in 2017, only 7 studies with relatively limited numbers of patients and controls have been published, which describe associations of microbiome patterns and ADHD in different cohorts applying diverse analytical methods. In the following, the main findings, special aspects and limitations of these studies, which are summarized in **Table 1**, will be described and discussed in the chronological order of publication.

**Table 1:** Characteristics of included studies.

Study	Study characteristics			Potential Confounders	N of Subjects			Results
First author, Year	Cases		Controls	Potential Confounders	Cases		Controls	
Aarts et al. (5)	Microbiome Sample: ADHD cases were diagnosed based on DSM-IV symptoms using the Schedule for Affective Disorder and Schizophrenia for School Age Children		Healthy participants & unaffected siblings of ADHAD patients & self-reported healthy volunteers	Not mentioned	N: 19 Age in years: 19.5(2.5) Males: 13 BMI: 23.8(4.1)		N: 77 included: - N: 17 healthy participants - N: 21 unaffected siblings of the ADHD Patients - N: 39 self- reported healthy volunteers Ages in years: 27.1 (14.3) Males: 41 BMI:23.0 (3.2)	
Aarts et al. (5)	FMRI Sample: from the above ADHD cohort follow up study: Children with ADHD no longer met the diagnostic criteria in adolescence or adulthood		Healthy & unaffected participants	Not mentioned	N: 24 Age in years: 20.3(3.7) Males: 18 BMI:22.8(3.5)		N:63 included: - N:39 unaffected siblings - N: 24: healthy controls Age in years: 21.3 (3.4) Males: 39 BMI:22.7(2.9)	Decreased ventral striatal response for reward anticipation in Patients with ADHD vs. controls (t(85)=2.1, p=.038)
Aarts et al. (5)	Microbiome and Imaging Analysis: From the above ADHD cohort		Healthy & unaffected participants		N:6 Age in Years:18.6(2.5) Males:4 BMI:22.1(4.4)		N:22 included: - N:13 unaffected siblings - N:9 healthy controls Age in Years: 21.1(3.3) Males: 13 BMI:23.4 (3.7)	No significant decreased ventral striatal response for reward anticipation in Patients with ADHD vs. Controls (t(26)= 0.2) Predicted CDT relative abundance significant associated with reward anticipation responses in ventral striatum (standardized beta: -0.42, p= 0.048)
Jiang et al. (6)	Juvenile Patients diagnosed with the Kiddle-SADS-Present and Lifetime Version (Kiddle-SADS-PL) Scale: semi-structured diagnostic interview conducted according to the DSM-IV classification system ADHD Symptom severity *via* parents CPRS		Healthy neurotypical Control Group recruited *via* advertisement and assessed with a semi-structured clinical interview to exclude individuals with a physical illness	Excluded confounders: Children with dietary habits Use of probiotics or antibiotics during the 2 months prior sample collection Apparent gastrointestinal symptoms, depressive or anxiety symptoms, obesity, common childhood atopic diseases &/or history of current use of ADHD drugs	N: 51 treatment naïve ADHD patients Age in Years: 8.47(0.47) Males:38 BMI:16.4 (2.02)		N: 32 Age: 8.5(8.47) Males: 22 BMI:16.09(2.02)	Significant decrease in the fractional representation of Faecalibacterium in children with ADHD vs. Controls Abundance of Faecalibacterium negatively associated with parental reports of ADHD Symptoms No significant difference in the alpha diversity between groups
Prehn-Kristensen et al. (7)		All patients met the DSM-IV criteria for ADHD Measures: German translation of the Revised Schedule for Affective Disorder and Schizophrenia for School-Age Children: Present and Lifetime Version (K-SADS-PL) CCBCL; German ADHD rating scale (FBB-HKS)	N:6 Patients fulfilled criteria for comorbid oppositional defiant disorder (ODD) N:10 Medicine for more than one year to treat ADHD Symptoms (9x Medikinet, 1x Equasym) N:9 medicine for at least 48h prior to sample		N:14 Age in years: 11.9 (2.5) BMI:19.0(3.9) Males: 14	N:17 Age in Years:13.1(1.7) BMI:18.0(2.5) Males: 17		Alpha diversity significant decreased in ADHD patient vs. controls (*P_shannon_*=.036) Beta diversity differed significantly between patients and controls (*P_ANOSIM_*) 0.033, *P_ADONIS_* =0.006, *P_betadisper_* =.002)	
Cheng et al. (8)		Diagnosed with DSM Criteria			N: 19099	N:34194		Desulfovibrio is associated with ADHD	
Wang et al. (9)		Patients with ADHD treated in the outpatient Department of a Child Psychiatry ADHD cases were diagnosed based on DSM-IV-TR trough structured interview based an K-SADS-E Dietary patterns through food frequency questionnaire	Children without ADHD Dietary patterns trough food frequency questionnaire	Excluded Confounders: Never taken any medications to treat ADHD; no psychiatric diseases or major physical illnesses No vegetarians or Patients who were currently taken probiotics or antibiotics	N: 30 Age in Years: 8.4 (1.7) Weight(kg): 30.7(10.2) Males: 23	N:30 Age in Years: 9.3(2.2) Weight(kg): 35.6(10.6) Males: 18		Gut microbiota communities in ADHD patients showed a significantly higher Shannon Index p=.0378) and Chao Index (p=.0351)as the controls Simpson Index was significant lower in ADHD patients	
Stevens et al. (10)		Micronutrient Treatment group Cases diagnosed with ADHD *via* ADHD Rating Scale IV (ADHD-RS-IV)	Placebo treatment Group Controls diagnosed with ADHD *via* ADHD Rating Scale IV (ADHD-RS-IV)	Not mentioned	N:10 Age: 9.3(1.3) BMI:16.6(3) Males: 10	N:9 Age: 10.29(1.9) BMI:19.39(2.9) Males: 9		OTUs significantly increased in the treatment group and no mean change in the placebo group (p:0.05) low abundance of Bifidobacterium was associated with a low ADHD-IV-RS score, which is contradictory to the general trend observed in the pre-RCT and placebo groups.	
Casas et al. (11)		ADHD assessed trough German parent- completed SDQ (10years) and self-completed version of SDQ (15 years)		Medication not mentioned Reporting bias Controlled confounders: parental education, indoor factors, e.g. indoor smoking	N:37 Male: 22 Hyperactivity/inattention - 10 Years old: 5 - 15 Years old: 37	N:189 Male: 95 Hyperactivity/inattention - 10 Years old: 18 - 15 Years old: 13		Early life bacterial diversity was inversely associated with hyperactivity/ inattention at age 10 [bacterial OTUs (medium vs low: aOR = 0.4, 95%CI = (0.2–0.8)) and Chao1 (medium vs low: 0.3 (0.1–0.5); high vs low: 0.3 (0.2–0.6)], fungal diversity was directly associated [Chao1 (high vs low: 2.1 (1.1–4.0)), Shannon (medium vs low: 2.8 (1.3–5.8)), and Simpson (medium vs low: 4.7 (2.4–9.3))] At age 15, only Shannon index was significantly associated with hyperactivity/inattention [bacteria (medium vs low: 2.3 (1.2–4.2); fungi (high vs low: 0.5 (0.3–0.9))]	

CRPS, Conners Parent Rating Scales; BMI(SD): SD, standard deviation; Age(SD): SD, standard deviation; CBCL, Child Behavior Checklist; FBB-HKS, Fremdbeurteilungsbogen für hyperkinetische Störung; DSM-IV-R, Diagnostic and Statistical Manual of Mental Disorders, Fourth Edition, Text Revision; K-SAD-E, Schedule for affective disorder and schizophrenia for school- age children, epidemiologic version; SDQ, Strength and Difficulties Questionnaire.

In 2017, Aarts et al. (5) published the first study characterizing the intestinal microbiome in Dutch ADHD patients by next-generation 16S rRNA gene sequencing. Nineteen ADHD and 77 control participants were included in the study. In a subset of 28 participants, independent of diagnosis, microbiome differences were interrogated in neural reward responses by functional magnetic resonance imaging (fMRI). Bacterial V3–V6 regions of 16s rRNA genes were analyzed by 454-pyrosequencing. Applying this method, no apparent differences in either alpha or beta diversity between the microbiome cases and controls were observed. However, a slight but significant increase of the genus Bifidobacterium (1.4- to 1.6-fold) was detected in ADHD patients. Predicting metabolic pathways from 16s rRNA-micobiome-analysis ADHD patients had more genes encoding cyclohexadienyl dehydratase activity responsible for the synthesis of dopamine precursors compared to controls. Of interest, during reward anticipation increased dopamine abundance was significantly linked to reduced ventral striatal fMRI responses. This enzyme is involved in the synthesis of phenylalanine, a precursor of dopamine and its increased abundance was. However, this was found to be independent of ADHD diagnosis and is thus not linked to disease development in that cohort. In this pioneering work, hypotheses on the functional role of the intestinal microbiota were derived from 16S rDNA-based high-throughput sequencing for the first time. However, the study exhibited limitations which might affect reliable conclusions: Previous medications of ADHD patients were not considered or reported in detail and the case and control groups for microbiome analyses were significantly different in age (27.1 vs 19.5 years in average). Also, the method of stool sampling was not reported.

Bypassing the potential interference of ADHD medication on microbial compositions, Jiang et al. (6) analyzed the gut microbiota composition in treatment-naive children with ADHD and its correlation with the severity of ADHD symptoms analyzing 51 juveniles with ADHD and 32 healthy controls in a Chinese cohort. Fecal samples from children without prior ADHD pharmacotherapies were collected in sterile plastic caps, immediately frozen at home at -20° C, and delivered to the laboratory in iceboxes within 30 min. Sequencing of 16S rRNA genes (V3 to V4 variable regions) on an Illumina MiSeq platform revealed no significant difference in alpha diversity between the ADHD and control groups but a significant decrease of *Faecalibacterium* in children with ADHD compared to healthy controls. Differences in dietary habits between high-fat Western and Chinese diet might explain deviating microbial patterns in ADHD patients compared to other studies. However, the authors of the study did not include questionnaires to account for nutritional differences.

In a German cohort, 14 preadolescent male ADHD patients (mean ages: 11.9 years) and 17 male controls (mean age 13.1 years) were analyzed regarding differences of microbial compositions by Prehn Kristensen et al. (7). Fecal samples of children and their parents were natively collected and stored at 4°C until preparation, before next-generation sequencing based analyses of V1 to V2 regions of 16S rDNA were conducted on the Illumina MiSeq platform. Ten out of 12 patients had been taken medicine for ADHD treatment. Medication had been discontinued 48 h prior to sample collection in 9 cases. Also, children and parents were interviewed using the K-SADS-PL schedule and dietary habits for intake of fast-food, meat/sausages/cold cuts, fruits/vegetables, or yoghurt and other milk products were inquired by a 4-point survey. ADHS children and controls did not differ in their dietary intake. Analysis of microbiome profiles did not reveal difference regarding their species richness, but Shannon indices were significantly decreased in ADHD. This decrease was also observed in microbiome profiles of mothers of ADHD patients, fathers did not show differences between both groups. This might indicate that microbial compositions were maternally passed on to their children. Also, a significant difference in beta diversity based on Bray Curtis dissimilarities was detected between ADHD and control children, which was based on differential abundances of different microbial taxa. LefSe analysis detected significant enrichment of genus *Bacteroides* and *Neisseria* spp. in ADHD children. However, the later did not differ in abundances but only in frequency between the study groups. *Prevotella* and *Parabacteroides* showed higher relative abundances in the control group. As a result, Prehn-Kristensen et al. suggest *Neisseria* and *Bacteroides* spp. as potential biomarkers for juvenile ADHD. The higher abundances of Bacteroides is in line with findings of Aarts et al. (5) and Wang et al. (9), who identified higher abundances in ADHS children and adolescents. The study of Prehn-Kristensen et al. however has several limitations. ADHD medication were administered in 9 out of 12 cases which could have a large impact on microbial compositions (12). Medication was discontinued 48 h before treatment, but this might not be sufficient to restore potential medication-driven persistent microbial patterns. Also, stool samples were collected without the use of stabilization reagents to preserve microbial compositions at the timepoint of sampling. In addition, non-stabilized native stool samples were stored at 4°C until preparation, which might facilitate compositional changes due to bacterial growth or degradation of nucleic acids within the samples.

Biological components of indoor air significantly effect human health by facilitating the development of allergic diseases such as asthma (13). To investigate the effect of microbial exposure in early life on the later development of ADHD, Casas et al. (11), sampled environmental samples from bedroom floor dust within the LISA (Lifestyle-related factors, Immune System and the development of Allergies in East and West Germany) birth cohort among 226 children at the age of three month. Indoor bacterial and fungal diversity was analyzed by collection of dust samples using a vacuum cleaner equipped with an ALK filter followed by subsequent analysis of bacterial 16S rRNA genes and fungal internal transcribed spacer (ITS) regions by high-throughput sequencing. At the ages of 10 and 15 years, hyperactivity/inattention behavior was evaluated using the Strength and Difficulties Questionnaire (SDQ). According to the questionnaire, a total of 23 children showing signs of ADHD at the age of 10 years were selected from the study population. The number of symptomatic children had raised to 50 individuals at the age of 15 years. To evaluate the associations between the home microbial diversity early in life and hyperactivity/inattention, fungal and bacterial alpha-diversity metrics (richness, Shannon, Simpson and Chao1 diversity indices) were calculated. The results of the study are indicating that a high load of a diverse bacterial environment may reduce the risk for a later development of ADHD, while a high number of fungal species might facilitate hyperactivity/inattention disorders. However, associations of bacterial and fungal diversity with ADHD prevalence were heterogeneous within the study. At age 10, bacterial richness was inversely correlated with ADHD prevalence while the number of fungal species were positively associated with a high prevalence. Diversity metrices for fungi showed an inverted U-shaped association with hyperactivity/inattention. This was not the case at age 15, where only the Shannon diversity index showed identical association characteristics for bacterial diversity as well as a negative correlation with fungal diversity.

Cheng et al. recently followed an integrated bioinformatic approach to explore potential relationships the gut microbiota and five different psychiatric disorders: ADHD, autism spectrum disorder, bipolar disorder, schizophrenia, and major depressive disorder (8). Applying gene set enrichment analysis (GSEA) they processed data from publicly available gnome-wide association studies (GWAS) originating from the Psychiatric GWAS Consortium. This extensive dataset comprised genomic data a total of 19,099 ADHD patients and 34,194 controls originating from the Psychiatric GWAS Consortium. To link gut microbiota profiles to ADHD-related genes or single nucleotide polymorphisms, the GSEA algorithm determines, which of those genes are significantly enriched in published datasets from genome-wide association studies of the human gut microbiota (GWASGM). Applying GSEA, the authors found significant associations of ADHD with the genus *Desulfovibrio* (P=0.031) and the order Clostridiales (P=0.034). However, they do not provide hypotheses on possible functional interrelationships in ADHD. In addition, *Desulfovibrio* was also significantly associated with any other psychiatric disorder tested in this study, so enrichment of *Desulfovirbio* abundance is probably not specific for ADHD. The authors provide an innovative strategy, one of the major drawbacks of the study is probably the limited knowledge about the interaction of the intestinal microbiota and host genetics and the narrow availability of datasets which are mainly based on the co-occurrence of genetic factors of the host and his inhabiting microbes. The study is referring to four different GWASGM studies (14–17). All subjects in these studies are aggregating to 2.815 individuals, which still might be underpowered with regard to the complexity of the underlying mechanisms (14–17).

In a double-blind, placebo controlled randomized control trial design, the group of Stevens et al, investigated the effect of broad-spectrum micronutrient supplementation on the stool microbiome of ADHD patients. Capsules containing placebo or a blend of vitamins, minerals, amino acids and antioxidants were administered to children in the age of 7–12 years (Treatment: n=10, Placebo: n=7) over a period of 10 weeks and fecal samples were collected using OmnigeneGut™ stool collection and stabilization kits (DNA Genotek). Micronutrient supplementation was associated with 50% responder rates, resulting in reduced impairment and improved attention as well as emotional regulation and aggression when compared to the placebo group (29% response). However, ADHD-IV-RS and CGAS metrics did not differ significantly in both groups. Microbiome sequencing based on 16S rDNA amplicons did not indicate changes of microbial alpha-diversity during micronutrient or placebo administration although the micronutrient group showed a higher species richness compared to the control group. On the compositional level, beta diversity did not change considerably in the course of the study within both groups. Also, metabolic capacities of the microbiome bioinformatically predicted from 16S rDNA data did not show significant changes. However, relative amounts of the phylum Actinobacterium (mainly comprising the genus *Bifidobacterium)* were significantly decreased post-micronutrient treatment (from 0.98% to 0.28%) accompanied with an increase of *Collinsella* abundances. These results are indicating a potential modulatory effect of micronutrients on putative probiotic bacterial species. However, the role of Bificobacteria in ADHD is contradictory and vague. While studies have shown protective effects of *Bifidobacterium longum* against psychiatric disorders including *ADHD* (18), the group of Aarts et al. reported an association of ADHD with higher relative abundances of *Bificobacterium* species (5). These opposite findings clearly illustrate the difficulties in interpretation of microbial patterns also considering a plethora of potential confounding factors like diagnostic heterogeneity of psychiatric disorders, low patient numbers, environmental factors, or the interpretation of compositional datasets in general.

In a Taiwanese study, the team around Wang et al. (9) compared fecal microbiota compositions and dietary habits in a group of 30 children diagnosed with ADHD (mean age: 8.4 years) with 30 healthy controls (mean age 9.3 years). ADHD diagnosis was based on the ADHD rating scale together with K-SADS-E interviews which have been conducted in both groups. Patients had not received any medication for ADHD treatment. Dietary habits were assessed using a food frequency questionnaire including 49 food items consumed from eight food groups. Microbial profiles based on 16S rDNA-sequencing of V3 to V4 variable regions were determined from stool samples using the Illumina MiSeq platform. Stool samples were natively collected and stored at -80°C within 24 h. Comparing alpha diversities of both study groups, ADHD patients showed a significantly higher bacterial diversity according to the Shannon and Chao diversity index. However, the Simpson index was significantly lower compared to the healthy control group. Microbiota profiles of ADHD and control groups were similar as they did not show significant differences of beta diversity according to the Principal Coordinates Analysis (PCoA) of UniFrac distances. Linear discriminant analysis effect size (LEfSe) revealed bacterial taxa which were correlated with ADHD symptoms. *Bacteroides coprocola* was significantly lower in the ADHD group, while *Bacteroides uniformis*, *Bacteroides ovatus,* and *Sutterella stercoricanis* were found to be enriched. In addition, the genus *Fusobacterium* was higher in the ADHD group (mean abundance 0.28%) than the controls (mean abundance = 0.02%), whereas members of the genus *Lactobacillus* were reduced in ADHD. This is in line with the findings of Aarts et al, who found also *Bacteroides uniformis* and *B. ovaus* being enriched in ADHD patients. Also, dietary intake varied between both study groups. ADHD-patients had a significantly higher intake of grains versus a lower intake of dairy and vitamin B2. *Sutterella stercoricanis* abundances correlated with the intake of dairy, nuts/seeds/legumes, ferritin, and magnesium, while *Bacteroides uniformis* was associated with the consumption of fat and carbohydrates. However, it has to be noted, that species level discrimination using short-read technologies for 16S rDNA-based sequencing is challenging. The genus *Bacteroides* share very similar 16S rDNA sequences, especially within the sequenced V3 and V4 variable regions. A non-accurate species classification will introduce bias in relative abundances of the respective species and will distort statistical interpretation. The authors did not validate their findings using alternative methods such as species-specific quantification by qPCR. Also, dietary habits were found to be different between ADHD and control groups. This aspect clearly illustrates particular critical issues of association-based microbiome studies. Differentially altered microbial compositions may only reflect variations in dietary intakes or other environmental factors.

Of special importance, methodological differences in the protocols applied in the published reports might strongly influence the microbiome composition analysis and thus the scientific conclusions. Recapitulating the results from recent studies investigating the role of the human microbiota in ADHD development and its pathophysiology, the majority of studies applied high-throughput 16S rDNA sequencing to investigate differential patterns in the microbiome of ADHD patients compared to healthy cohorts. The heterogeneity in accurate neuropsychiatric diagnosis of ADHD and a vast number of confounding environmental factors which are known to shape microbial compositions in the intestine like nutritional intake, ADHD-targeted or other medication as well as elevated physical activity of ADHD patients compared to controls might impede clear associations of microbial patterns. In addition, methodological obstacles are impeding identification of accurate and reproducible microbial compositions. To date, microbiome sequencing is still lacking standardization and implementation of precise quality controls, which lead to an overall low comparability of microbiome studies (19). Variations in implemented methods or protocols might strongly influence the microbiome analysis results within study cohorts: Inappropriate preservation of microbial patterns in stool samples during sampling and storage, differences in DNA-isolation and purification protocols, different species coverage of PCR primer pairs applied, the lack of spike-in materials to be used as process control and evaluation of absolute bacterial abundances in stool samples as well as inaccuracies of bioinformatic pipelines resulting in imprecise taxonomic classification of sequencing reads or an overestimation of spurious operational taxonomic units (20). These factors contribute to conflicting and often non-reproducible results in ADHD studies. Further efforts in standardization are needed to reduce the risk of methodological bias within all preanalytical and analytical steps, beginning from suitable strategies for stool sampling to bioinformatic analysis.

## Conclusions

Despite the fact that ADHD is the most common neurodevelopmental disorder worldwide affecting more than 7% of the children of whom more than half continue to have the disorder in adulthood, relatively little is known on the contribution of microbiome shifts to ADHD predisposition and development so far. Due to high variability in cohorts, important methodological differences of microbiome analysis as well as frequently very low number of included patients (167 clinically characterized patients in total included in 6 studies) it is too early to define predictive microbiome biomarkers for ADHD or draw conclusions about underlying microbiome-mediated pathomechanisms of the disease. In addition, the analysis of the taxonomic composition of the microbiome alone is a clear limitation and frequently an inappropriate correlate with host phenotype, which might be better predicted by prevalent microbial molecular function or genetic differences on the sub-species level of bacteria. This conception was the basis for the development of the second phase of the human microbiome project, HMP, the Integrative HMP (iHMP or HMP2) (21), which was aims to analyze the host–microbiome interplay, including metabolism, immunity, and additional molecular mechanisms driving host cell physiology, to develop more integrated picture of host–microbe interactions over time. This multi-omics initiative aims to increase the data base as well as standardize protocols for the microbiome research community, to better evaluate the mechanistic relationship between host and microbiome in the future. An ADHD-targeted multicenter study using multiple complementary approaches to define the functional role of the gastrointestinal microbiome for ADHD longitudinally and to provide protocols, biospecimens, and data for future work could help to solve the most interesting questions regarding the gut-brain-axis in ADHD possibly leading to novel therapeutic strategies.

## Author Contributions

AG, JG, and AH contributed conception and design of the study, performed the database searches and wrote parts of the manuscript. All authors contributed to the article and approved the submitted version.

## Funding

Funded by the Deutsche Forschungsgemeinschaft (DFG, German Research Foundation), project ID B13 - TRR221” provided to AG.

## Conflict of Interest

The authors declare that the research was conducted in the absence of any commercial or financial relationships that could be construed as a potential conflict of interest.
